# Activin in the Brain Modulates Anxiety-Related Behavior and Adult Neurogenesis

**DOI:** 10.1371/journal.pone.0001869

**Published:** 2008-04-02

**Authors:** Hiroshi Ageta, Akiko Murayama, Rika Migishima, Satoshi Kida, Kunihiro Tsuchida, Minesuke Yokoyama, Kaoru Inokuchi

**Affiliations:** 1 Mitsubishi Kagaku Institute of Life Sciences (MITILS), Machida, Tokyo, Japan; 2 Japan Science and Technology Corporation (JST), CREST, Kawaguchi, Japan; 3 Graduate School of Environment and Information Sciences, Yokohama National University, Yokohama, Japan; 4 Department of Bioscience, Tokyo University of Agriculture, Setagaya-ku, Tokyo, Japan; 5 Division for Therapies Against Intractable Diseases, Institute for Comprehensive Medical Science, Fujita Health University, Aichi, Japan; 6 Brain Research Institute, Niigata University, Niigata, Japan; Wellcome Trust Sanger Institute, United Kingdom

## Abstract

Activin, a member of the transforming growth factor-β superfamily, is an endocrine hormone that regulates differentiation and proliferation of a wide variety of cells. In the brain, activin protects neurons from ischemic damage. In this study, we demonstrate that activin modulates anxiety-related behavior by analyzing ACM4 and FSM transgenic mice in which activin and follistatin (which antagonizes the activin signal), respectively, were overexpressed in a forebrain-specific manner under the control of the αCaMKII promoter. Behavioral analyses revealed that FSM mice exhibited enhanced anxiety compared to wild-type littermates, while ACM4 mice showed reduced anxiety. Importantly, survival of newly formed neurons in the subgranular zone of adult hippocampus was significantly decreased in FSM mice, which was partially rescued in ACM4/FSM double transgenic mice. Our findings demonstrate that the level of activin in the adult brain bi-directionally influences anxiety-related behavior. These results further suggest that decreases in postnatal neurogenesis caused by activin inhibition affect an anxiety-related behavior in adulthood. Activin and its signaling pathway may represent novel therapeutic targets for anxiety disorder as well as ischemic brain injury.

## Introduction

Anxiety disorder represents one of the most common mental illnesses [1]–[3]. Recently, disturbance in adult hippocampal neurogenesis was proposed to underlie anxiety-like behavior in rodents [4], [5]; however, molecular mechanisms that link hippocampal neurogenesis to anxiety disorder remains poorly understood.

Activin, a member of the transforming growth factor-β superfamily, is an endocrine hormone that regulates differentiation and proliferation of a wide variety of cells [6]. In the brain, activin receptor ActRII is highly expressed in forebrain region [7], [8], and its scaffold protein ARIP/S-SCAM is also localized in synaptic region [9], [10]. Furthermore, activin protects neurons from ischemic damage [11], and its expression is upregulated by neuronal activity [12], [13]. Recently, we showed that activin modulates dendritic spin morphology that is important for synaptic plasticity in the hippocampus [14], [15].

In this study, we generated and analyzed transgenic mice in which activin function in the forebrain is either suppressed or enhanced. We found that the activin activity in the adult forebrain influences locomotor activity, anxiety-related behavior, and hippocampal neurogenesis.

## Results

**Figure 1:**
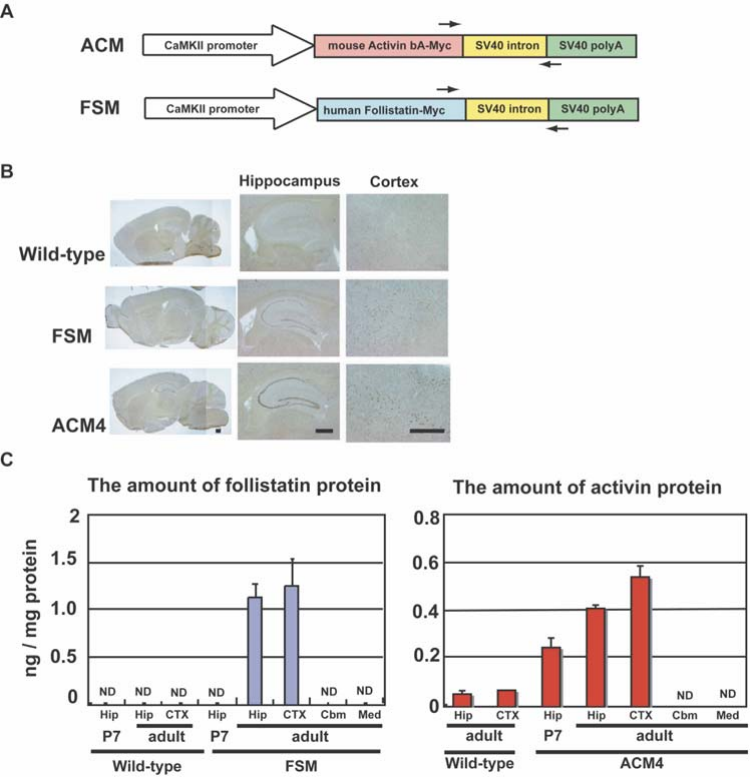
Generation of transgenic mice and expression analysis of the transgene. (A) Schematic representation of transgene constructs. Narrow arrows indicate the location and direction of RT-PCR primers (Figure S2). (B) Photographs of typical *in situ* hybridization are shown. DIG-labeled cRNA probe corresponding to the SV40 polyA sequence was hybridized to sagittal sections of the brain from 16–22 week-old wild-type, FSM and ACM4 mice. Scale bars, 500 µm. (C) Follistatin and activin protein levels in the hippocampus (Hip), cortex (CTX), cerebellum (Cbm) and medulla (Med) in wild-type, FSM and ACM4 mice measured by ELISA. Results are shown as mean±s. e. m. (n = 4) Except for activin in CTX in wild-type (n = 1). Adult indicates 16–22 weeks-old. P7, postnatal day 7.

We explored the role activin plays in anxiety-related behavior using a transgenic mouse model that overexpresses activin or follistatin, an activin-inhibitory protein, in a forebrain-specific manner. Disturbance of activin signal during the developmental stage causes a lethal phenotype in mammals [16], [17]. To achieve postnatal, forebrain-specific expression, the αCaMKII promoter was used to drive expression of a transgene (**Fig. 1A**) [18], [19]. We microinjected activin and follistatin transgenes into 549 and 1183 fertilized eggs, and obtained 42 and 55 weaned mice, respectively. From these, two lines of activin transgene-integrated mice (designated ACM3 and ACM4) and one line of follistatin transgene-integrated mice (designated FSM) were obtained. Transgene-integrated mice were generated in 1% of microinjected fertilized eggs [20]. This low efficacy may have been caused by unexpected transgene expression in various tissues during the embryonic stage, because the activin signal is important for normal development. In contrast to previously generated activin- or follistatin-transgenic mice [21]–[23], these heterozygous transgenic mice were fertile and bred healthily, and their body weight (data not shown) and muscular strength were normal when compared to their wild-type littermates (**Figure S1**). Since ACM3 mice showed phenotypes similar to ACM4 mice in behavioral experiments, we hereafter describe the phenotypes of ACM4 and FSM mice.

*In situ* hybridization analyses of brain sections revealed that transgene expression was restricted to the forebrain such as the hippocampus and neocortex in the adult brain (**Fig. 1B**). ELISA analyses also showed forebrain-specific expression of activin and follistatin in ACM4 and FSM adult mice, respectively (**Fig. 1C**). Low level of endogenous activin was detected in the hippocampus and neocortex in wild-type mice. Follistatin level in FSM was enough to antagonize this level of activin activity [24]. Follistatin was not detected in the infant hippocampus of FSM mice (**Fig. 1C**). RT-PCR revealed that follistatin- and activin-transgene were not expressed in peripheral tissues including, heart, lung, spleen, liver and kidney (**Figure S2**). Nissel staining showed no apparent structural abnormality in the hippocampus of each transgenic mice (**Figure S3**).

**Figure 2:**
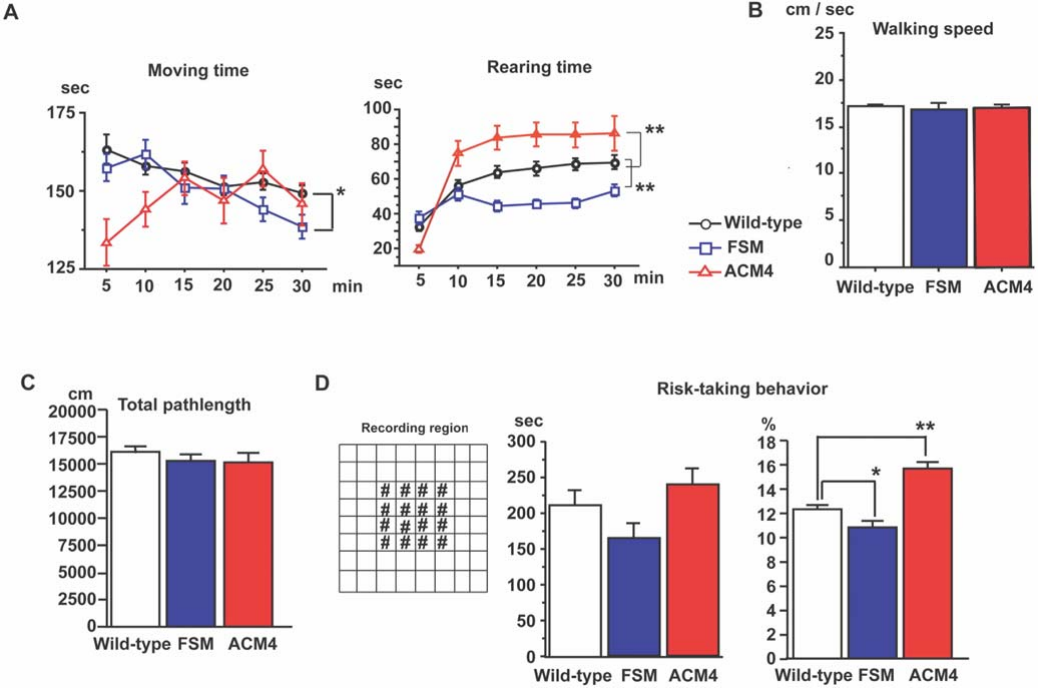
Activin protein levels in the brain influence locomotor activity. (A) Statistical analyses of the open field test showing time spent in locomotion and rearing [wild-type littermates (black circles), n = 34; FSM (blue squares), n = 18; ACM4 (red triangles), n = 11]. Each plot represents an average of 5 minutes. *, *p*<0.05; **, *p*<0.001; Fisher's test. (B and C) Statistical analyses of walking velocity (B) and total pathlength (C) during 30 minutes of open field test. (D) Risk taking behavior test. Left panel, overhead view of the box used for open field test. #, area defined as a center region. Mid panel, time spent in the center region during the 30 minutes of open field testing. Right panel, the percentage pathlength in the center region. Results are shown as mean±s. e. m.

Open field tests were performed on transgenic mice to investigate locomotor activity (**Fig. 2**). FSM mice showed a decrease in time spent in locomotion and rearing when compared with wild-type littermates. In contrast, ACM4 mice showed a significant increase in rearing time. There was no genotype effect in the walking speed (**Fig. 2B**) and the total pathlength (**Fig. 2C**), indicating that walking ability of FSM and ACM4 mice was normal. These results indicate that the level of functional activin in the brain is related to general locomotor activity in a novel environment.

**Figure 3:**
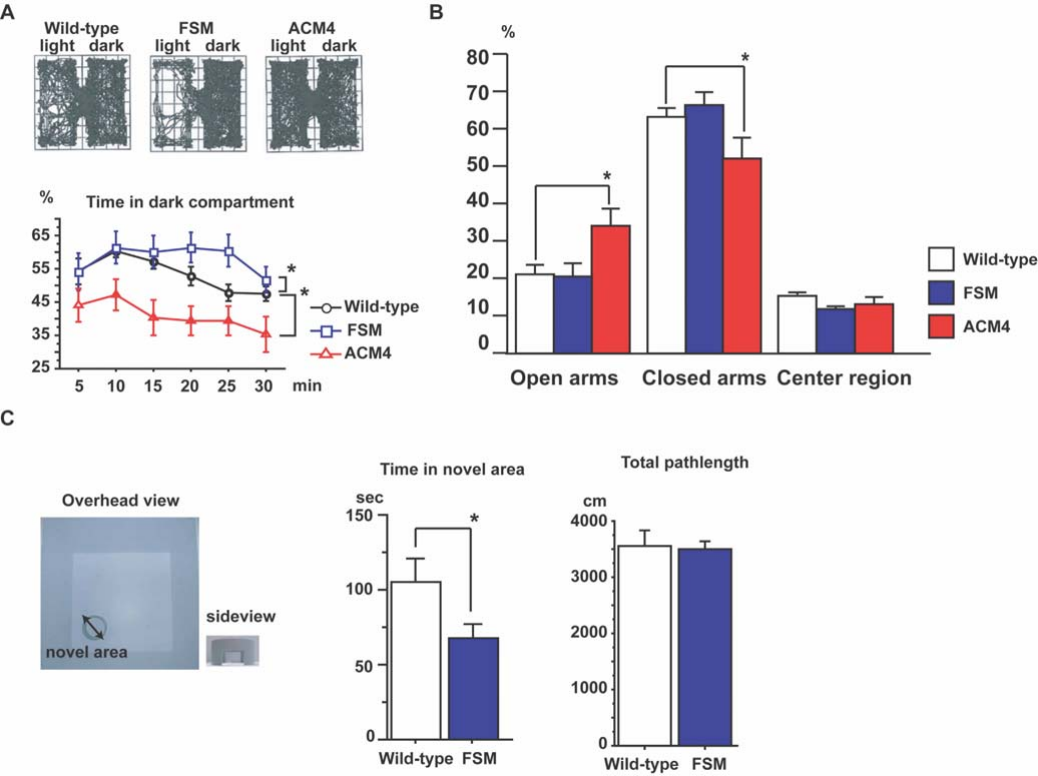
Activin protein levels in the brain modulate anxiety-related behavior. (A) Upper panels, typical traces in the light and dark test for each genotype. Lower panels, time (%) spent in the dark compartment was measured over 30 min. Wild-type littermates (black circles), n = 34; FSM (blue squares), n = 18; ACM4 (red triangles), n = 11. *, *p*<0.05, Fisher's test. (B) Statistical analyses of elevated plus-maze. Wild-type littermates, n = 26; FSM, n = 7; ACM4, n = 11. *p* values indicate ANOVA for genotype effect. *, *p*<0.05; t-test. (C) Left panels shows the apparatus used for the novel-area accessing test. Arrows indicate points of entry to the cylinder. Small picture shows side view of the cylinder. Bar graphs show time spent in novel area (inside cylinder) and distance traveled during 10 min testing. *, *p*<0.05, t-test. Results are shown as mean±s. e. m.

In open field tests, the amount of time spent in the center of the field is strongly correlated with an animal's level of anxiety, a characteristic called risk-taking behavior [25], [26]. FSM mice showed decreased performance in risk-taking behavior (**Fig. 2D**), while ACM4 mice showed increased performance. To further assess these differences, a light and dark choice test and elevated plus-maze test were conducted. In the light and dark test, ACM4 mice accessed the lighted compartment significantly more often than wild-type littermates (**Fig. 3A**), however, FSM mice spent significantly more time in the dark compartment as compared to wild-type littermates. In the elevated plus-maze test, ACM4 mice spent significantly more time in the open arms of the testing apparatus than did wild-type and FSM mice (**Fig. 3B**). FSM mice showed no significant change in phenotype for this test.

We next designed and performed an original behavioral test to measure anxiety levels (**Fig. 3C**), based on the observation that mice generally prefer novel objects encountered in a familiar place [27]. In this test, mice were placed in a closed box on the first day to become familiar with the box. On the second day mice were placed in the same box to which a cylinder with two entrances (novel area) had been added. FSM mice spent significantly less time accessing the novel area as compared to wild-type mice, while the total distance they traveled during the test was normal. This suggests that higher anxiety in FSM mice resulted in lower access to the novel area. Taken together, the level of functional activin in the brain modulates anxiety-related behavior. Finally, no depressive behavior was observed in FSM mice in the forced swimming test (**Figure S4**).

Adult neurogenesis is the production of new neurons in areas of the adult brain including the subventricular zone (SVZ) and subgranular zone (SGZ) of the hippocampus [28]. This formation of new neurons plays a number of physiological roles including damaged neuron replacement[29], [30], memory formation [31], [32] and response to stress [33]. Moreover, some reports have recently shown that neurogenesis is involved in depression [34], [35].

**Figure 4:**
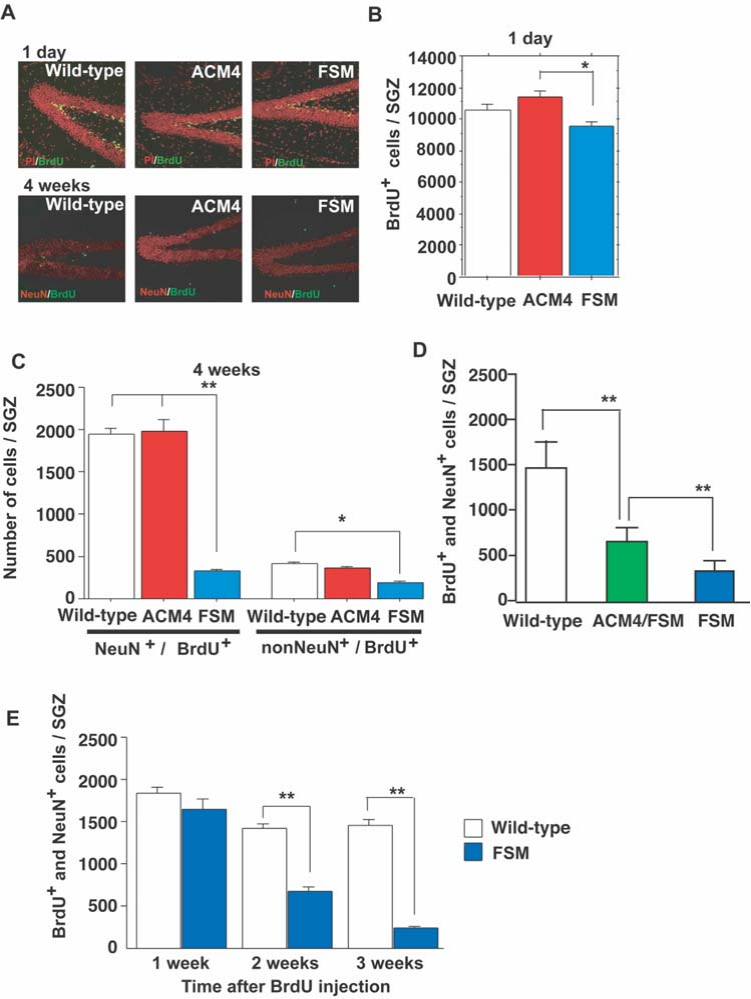
Activin signal is essential for survival of newly generated neurons. (A) BrdU positive cells (green) in the SGZ. Mice were sacrificed 1 day (upper panels) or 4 weeks (lower panels) after BrdU administration. At 4 weeks, most BrdU-positive cells were co-labeled with NeuN (red), a marker for mature neurons. Propidium iodide (PI, red) was used as a nuclear marker. (B) Number of BrdU-positive cells in the SGZ. The Y-axis indicates the number of BrdU-positive cells of the entire hippocampus. ACM4 had more BrdU-positive cells than FSM (wild-type, n = 21 animals; ACM4, n = 6 animals; FSM, n = 10 animals). (C) Number of BrdU-positive cells differentiated to neurons (NeuN^+^/BrdU^+^) or differentiated to another cell type (non-NeuN^+^/BrdU^+^) in the SGZ (wild-type, n = 25; ACM4, n = 14; FSM, n = 9). Animals were sacrificed 4 weeks after BrdU administration. (D) Number of BrdU- and NeuN-double positive cells in the SGZ (wild-type, n = 32; ACM4/FSM, n = 9; FSM, n = 14). Animals were sacrificed 4 weeks after BrdU administration. (E) Number of BrdU/NeuN-double positive cells in the SGZ. Animals were sacrificed 1, 2, or 3 weeks after BrdU administration. At the 2-week stage (wild-type, n = 7; FSM, n = 8), but not at the 1-week stage (wild-type, n = 7; FSM, n = 9), the number of double positive cells was significantly decreased in FSM mice compared with wild-type littermates. Error bars indicate the s. e. m. *, *p*<0.05; **, *p*<0.01; t-test.

We therefore examined adult neurogenesis in hippocampal SGZ of FSM and ACM4 mice (**Fig. 4**) using 5-bromodeoxyuridine (BrdU)-labeling experiments. Transgenic mice were injected with BrdU (75 mg/kg body weight) three times per day for three consecutive days. Mice were sacrificed either 24 h or 4 weeks after the final injection day. BrdU is incorporated into genomic DNA by cells at S-phase, therefore, by staining with a neuronal marker (NeuN) and an anti-BrdU antibody, newly generated neurons were easily detected. A significant difference between FSM and ACM4 mice was observed in the number of SGZ BrdU-positive cells after 24 h (**Fig. 4A, B**). No significant difference, however, was observed between transgenic mice and wild-type littermates, indicating that the number of neuronal progenitor cells and the rate of BrdU incorporation into progenitor cells in transgenic mice were essentially normal. At the 4-week stage, however, the number of BrdU- and NeuN-double positive cells in FSM mice was markedly decreased (**Fig. 4C**). This reduction was partially rescued by crossing with ACM4 (**Fig. 4D**). These results indicated that the level of activin in the brain is crucial for the maturation and maintenance of newly generated neurons.

The decrease in BrdU- and NeuN-double positive cells at the 4-week stage may be attributed to a decrease in the survival rate of newly formed neurons or a decrease in the rate for neuronal differentiation of new cells. Therefore, the change in the number of BrdU- and NeuN-double positive cells following BrdU injections (**Fig. 4E**) was monitored at various developmental stages. The number of BrdU- and NeuN-double positive cells was normal at the 1-week stage in FSM mice, suggesting a normal differentiation rate. However, a marked decrease was observed in the number of BrdU- and NeuN-double positive cells at 2- and 3-week stages compared with wild-type littermates. Therefore, in FSM mice, the survival of newly generated neurons is significantly decreased. This indicates that activin signal is essential for the maintenance of newly generated neurons. Activin overexpression did not enhance the number of BrdU- and NeuN-double positive cells at 4 weeks, suggesting that activin overexpression is not sufficient for enhancement of adult neurogenesis (**Fig. 4C**).

Taken together, FSM and ACM4 mice showed opposite phenotypes in behavior. Furthermore, decrease in neurogenesis in FSM mice was partially rescued in FSM/ACM4 double transgenic mice. These results strongly suggest that the observed effects of overexpression, either follistatin or activin, are not positional transgene effects such as insertional mutations.

## Discussion

There is a marked overlap and coincidence between anxiety and depression [1]–[3]. Depression is a serious disorder in our current society. Many popularly prescribed antidepressant drugs work to modulate monoamine neurotransmission, and take six to eight weeks to exert their effects [3]. Each drug is efficacious in only 60–70% of patients. Therefore, a conceptually novel antidepressant that acts rapidly and safely in a high proportion of patients would be highly advantageous [3]. We show here that activin in the forebrain bi-directionally influences anxiety-related behavior. Depression is usually seen in anxiety patients, and anxiety is often reported in depressed patients [1]–[3]. A recent paper by Dow *et al.* showed that activin infusion into the hippocampus produced an antidepressant-like effect [36]. Therefore, the level of activin in the hippocampus modulates both depressive and anxiety-related behavior. Therefore, activin may represent a new contributing factor for the modulation of anxiety. The transgenic mice used in this study may be useful for screening compounds in the development of new mechanistically-novel anti-depressant drugs.

## Materials and Methods

### Transgene construction and generation of transgenic mice

Coding region for mouse activin or human follistatin was amplified by PCR using specific PCR primers designed to add a Kozac sequence at the N-terminus and a myc-tag sequence at the C-terminus. The resulting cDNAs were cloned into pcDNAI (Invitrogen) to append an SV40 intron/polyadenylation signal at the 3′-end. These constructs were inserted into the Not I site of pMM403 vector (kindly provided by Dr. M. Mayford) [18] which contains the alpha calcium/calmodulin-dependent protein kinase II (αCaMKII) promoter to generate pCaM-activin-Myc and pCaM-follistatin-Myc plasmids. *Sfi* I fragments were isolated from pCaM-activin-Myc or pCaM-follistatin-Myc and microinjected into the pronuclei of one-cell embryos of C57BL/6J mice to produce transgenic mice [37]. Microinjected embryos were transferred to the oviducts of pseudo-pregnant females. Founder transgenic mice were identified by Southern blot analyses and PCR analysis with genomic DNA prepared from tail, and bred with C57BL/6J mice. Forward (f) and reverse (r) PCR primers for genotyping were as follows: ACM, f-5′-CACCCACTAGCCGTTACCAT-3′and r-5′-ATCCTCTCAGCCAAAGCAAG-3′; FSM, f-5′-GAGGTAGGAAGAGCGATGAT-3′and r-5′-CTCCATCATTCCCACAGAGA -3′. C57BL/6J mice were purchased from Clea Japan Inc. (Tokyo, Japan)

### ELISA analysis

After removal of the brain, each neuronal tissue (hippocampus, cortex, cerebellum and medulla) was quickly dissected out. Tissues were homogenized in lysis buffer [5 mM Tris-HCl, pH 8.0, 0.32 M sucrose, protease inhibitor cocktail (Sigma)], and homogenates were centrifuged at 20,000×g at 4°C for 10 min. Supernatant was collected and assayed for quantification of total protein with the BCA™ Protein Assay Kit (Pierce). Follistatin and activin levels were assayed by using commercial ELISA kits (Human Follistatin Immunoassay, AN'ALYZA and Activin-A ASSAY, Oxford Bio-Innovation, respectively).

### Neurogenesis

Male mice at 5 weeks-old received daily intraperitoneal injections of BrdU (Sigma) in 0.9% NaCl solution (75mg/kg, three times per day for three days). Animals were sacrificed with an overdose of anesthetics and perfused transcardially with 0.9% saline followed by 4% paraformaldehyde (PFA) in PBS. Brains were stored in fixative (4% PFA in PBS) for 3 h at 4°C, and incubated overnight in 30% sucrose, and then immersed in dry ice powder. A cryostat was used to collect sagittal sections of 14-μm thickness. The sections were boiled for 10 min, and then treated with 2M HCl for 30 min. After rinsing in 0.1 M boric acid (pH 8.5) for 10 min, tissues were incubated in 1% H_2_0_2_ in PBS for 30 min, and then blocked with 0.1% BSA and 3% goat serum in PBS containing with 0.1% Tween20 (PBST) at room temperature for 1 h. After blocking, tissues were incubated with blocking solution containing rat monoclonal anti-BrdU (1:250; Accurate) and mouse monoclonal anti-NeuN (1:200; Chemicon) antibodies. Sections were then incubated with anti-rat IgG-FITC and anti-mouse IgG-Rhodamine.

For quantification, three consecutive serial sections at section-interval 13 were used for counting BrdU-positive cells though an ×40 objective (BX41, OLYMPUS) in a genotype-blinded manner. Total number of BrdU-positive cells was obtained by multiplying the number of BrdU-positive cells counted in all the sections by 13/3. Figures were imaged by confocal microscopy operated under manual control (LSM5 PASCAL, ZEISS).

### Animal care and data analysis

All procedures involving mice were performed in compliance with National Institutes of Health guidelines and were approved by the Animal Care and Use Committee of Mitsubishi Kagaku Institute of Life Sciences, MITILS. We abided by MITILS guidelines on animal husbandry. All behavior experiments were conducted in a blinded fashion on male, heterozygous transgenic mice and their wild-type littermates (5–6 months). Two weeks before behavioral analysis, animals were housed individually in plastic cages and maintained on a 12:12-h light:dark cycle. Food and water were provided *ad libitum*. For five days before behavioral analysis, the mice were handled daily. Statistical analyses were conducted using StatView (Abacus Concepts). Values were expressed as mean±s. e. m..

### *In situ* hybridization

To detect transgene expression the SV40 poly A signal sequence, which is found in each transgene, was used as an antisense probe (Fig 1A). To prepare the probe, this region was subcloned into pBluescript II (Stratagene) to generate pSV40. pSV40 was digested with BamHI to generate DNA template for *in vitro* transcription of antisense cRNA probe. Digoxigenin-labeled antisense cRNA probe was produced by transcription with T7 polymerase. For hybridization experiments, animals were sacrificed with an overdose of anesthetics, and the brain was dissected and immediately frozen on dry ice. Cryostat sections (20-μm thickness) were cut and mounted onto polylysine-coated glass slides. Sections were air-dried and stored at −80°C until use in hybridization. *In situ* hybridization was carried out as described previously [38].

### Behavioral analysis

Behavioral experiments were basically carried out as described previously [39]. In the open field test, spontaneous locomotor activity was measured in a square arena (50×50×30 cm; Muromachi Kikai, Japan) by using a device outfitted with photo-beam detectors for monitoring horizontal and vertical activity, namely, distance traveled, time spent in locomotion, rearing counts and time in rearing. For statistical analysis on the percentage pathlength in the center region, we used ImageJ program (developed at the U. S. National Institutes of Health, and available on the Internet at http://rsb.info.nih.gov/ij/), Mice were allowed to explore freely while data was collected for 30 min. In the light and dark test, the square arena was divided into light and dark compartments. Data was collected as mice were allowed to freely traverse the arena among the two compartments for 30 min.

The elevated plus-maze comprises areas of two opposing open arms (25×5×0.5 cm) and two opposing enclosed arms (25×5×15 cm), connected by a central platform (O'HARA & Co, Japan). Mice were placed in the center area, and allowed to explore for 10 min. Their activity was recorded by video camera. Results were analyzed on a Macintosh computer using Image EP2.13 (O'HARA & Co), modified software of the public domain NIH Image program (developed at the U. S. National Institutes of Health, and available on the Internet at http://rsb.info.nih.gov/nih-image/).

In the novel-area accessing test (**Fig. 3C**), mice were placed in the center of a box (60×60×50 cm) for 10 min on the first day in order to habituate the apparatus. The next day mice were placed in the same box to which a novel cylinder (13 cm in diameter) with two entrances (6×3.5 cm) was added. Mice were allowed to explore for 10 min, while their activity was recorded by video camera. Results were analyzed on a Macintosh computer using Image OEC 1.02r1 (O'HARA & Co), a modified software of the public domain NIH Image program.

## Supporting Information

Figure S1 Traction test indicates that grip strength was comparable between transgenic mice and wild-type littermates. Forelimb grip strength was quantitatively assessed using a spring strain gauge (O'HARA & Co., Japan). Animals held by the tail grasped a wire netting and were gently pulled away from the bar with a smooth steady pull until they released the wire netting. The Y-axis indicates grip strength (g).
(0.16 MB TIF)

Figure S2 Transgene expression was not detected in the internal organs of FSM and ACM4 mice. RT-PCR-based identification of transgene expression was carried out in various internal organs. Transgene plasmid DNA and total cellular RNA prepared from cortex of each transgenic mice line served as a positive control. Water and total cellular RNA from cortex of wild-type mice served as a negative control. Actin gene was used as an internal control. Unspliced products were detected at the upper position of mature and spliced product when transgene plasmid DNA or ACM4's cortex RNA were used as a template. To rule out the possibility that genomic DNA was amplified, we performed RT-PCR without reverse transcription (middle row panels, RT-), which showed no signals.
(0.56 MB TIF)

Figure S3 Nissl staining of the coronal brain section from wild-type, FSM and ACM4. Lower panels, high magnification images of the hippocampus. Scale bar, 500 μm. Method. Animals were sacrificed with an overdose of anesthetics, and the brain was dissected and immediately frozen on dry ice. Cryostat sections (18-μm thickness) were cut and mounted onto polylysine-coated glass slides. Sections were air-dried and stored at −80°C until use. Slides were immersed in the 10% formalin solution for 30 min at 4°C for the fixation, and washed twice with PBS for 15 min at room temperature. Slides were then stained with 0.1% Cresyl Violet for 10 min. They were differentiated in H_2_O for 3–5 min and then dehydrated through 70%, 95%, 100% and 100% alcohol. They were then put in xylene and cover-slipped.
(0.76 MB TIF)

Figure S4 Analysis of forced swimming test [wild-type littermates (black circles), n = 18; FSM (blue squares), n = 8] on day 2. The immobilizing time (sec) was plotted for each minute. No significant genotype effect was observed for FSM. On day 1, mice were placed in a container with water at a depth of 20 cm (23–25°C) for 15 min. and forced to swim as they were unable to touch the bottom with their hind limbs. On day 2, the mice were placed back into 20 cm deep water for 5 min. When mice were unable to avoid the forced swimming, they exhibited immobility. Immobility was monitored by infra-red detector (CompACT FSS, Muromachi Kikai).
(0.13 MB TIF)
